# Dynamic transcriptional profiling provides insights into tuberous root development in *Rehmannia glutinosa*

**DOI:** 10.3389/fpls.2015.00396

**Published:** 2015-06-10

**Authors:** Peng Sun, Xingguo Xiao, Liusheng Duan, Yuhai Guo, Jianjun Qi, Dengqun Liao, Chunli Zhao, Yan Liu, Lili Zhou, Xianen Li

**Affiliations:** ^1^Center for Medicinal Plant Cultivation, Institute of Medicinal Plant Development, Peking Union Medical College, Chinese Academy of Medical SciencesBeijing, China; ^2^State Key Laboratory of Plant Physiology and Biochemistry, College of Biological Sciences, China Agricultural UniversityBeijing, China; ^3^Department of Agronomy, College of Agriculture and Biotechnology, China Agricultural UniversityBeijing, China

**Keywords:** *Rehmannia glutinosa*, tuberous root, development, transcriptome, RNA-seq

## Abstract

*Rehmannia glutinosa*, an herb of the Scrophulariaceae family, is widely cultivated in the Northern part of China. The tuberous root has well-known medicinal properties; however, yield and quality are threatened by abiotic and biotic stresses. Understanding the molecular process of tuberous root development may help identify novel targets for its control. In the present study, we used Illumina sequencing and *de novo* assembly strategies to obtain a reference transcriptome that is relevant to tuberous root development. We then conducted RNA-seq quantification analysis to determine gene expression profiles of the adventitious root (AR), thickening adventitious root (TAR), and the developing tuberous root (DTR). Expression profiling identified a total of 6794 differentially expressed unigenes during root development. Bioinformatics analysis and gene expression profiling revealed changes in phenylpropanoid biosynthesis, starch and sucrose metabolism, and plant hormone biosynthesis during root development. Moreover, we identified and allocated putative functions to the genes involved in tuberous root development, including genes related to major carbohydrate metabolism, hormone metabolism, and transcription regulation. The present study provides the initial description of gene expression profiles of AR, TAR, and DTR, which facilitates identification of genes of interest. Moreover, our work provides insights into the molecular mechanisms underlying tuberous root development and may assist in the design and development of improved breeding schemes for different *R*. *glutinosa* varieties through genetic manipulation.

## Introduction

*Rehmannia glutinosa*, commonly known as the Chinese foxglove (Scrophulariaceae), is native to China, Japan, and Korea. The Chinese medical classic “Shen Nong Ben Cao Jing” (the earliest monograph on Chinese materia medica) lists *R*. *glutinosa* as a “top grade” herb (Yang, 2007). Traditional Chinese medicine uses three types of tuberous roots, namely, fresh, dried, and treated, to nourish the *Yin* (the negative, dark, and feminine, according to Chinese philosophy), abate pathogenic heat, strengthen the body, and replenish vitality (Li, 2014). Modern pharmacological studies indicate that Rehmannia extracts exert broad-spectrum pharmacological effects on various human systems such as the circulatory, endocrine, cardiovascular, nervous, and immune systems (Zhang et al., 2008). There has been a surge in the use of Rehmannia in treating ailments such as angiocardiopathy, diabetes, hypertension, vascular dementia, cerebral infarction, urinary tract problems, and menstrual disorders (Li, 2014).

*R*. *glutinosa* has been commercially cultivated in China for over a thousand years, and several high yielding cultivars such as “Beijing No. 1” and “85-5” were developed by Chinese breeders through cross breeding and selection. The cultivar “85-5,” which was developed in the 1980s, is characterized by big tuberous roots and high yield. It has an average fresh weight of 24.5 tons per hectare (Li et al., 2007), which is comparable with that of sweet potato, a globally important food crop. Under high-density planting and plastic film sheeting conditions, the yield of “85-5” can even reach 95.0 tons per hectare (Li et al., 2009a). Rehmannia has a high yield potential; it suffers a significant reduction in tuberous root yield due to viral infections and replanting diseases (Wen et al., 2001). Replanting occurs during consecutive plantings and causes a severe decline in the productivity and quality of the tuberous products (Liu et al., 2006; Zhang et al., 2011). Researchers have studied the anatomical and hormonal changes associated with induced tuberous root formation *in vitro* to combat diseases and improve its productivity (Xue et al., 2004a,b). The molecular basis of replanting diseases had piqued the interest of researchers (Yang et al., 2011, 2014; Li et al., 2013); however, research studies on this particular area are limited. Understanding of the molecular process of tuberous root development will greatly facilitate the genetic improvement of Rehmannia in terms of yield, quality, and medicinal values.

Despite the extensive research efforts to identify genes involved in tuberous root development, the molecular mechanism underlying tuberous root development in Rehmannia remains unclear (Sun et al., 2010). The limited genomic information on Rehmannia consists of 1301 EST sequences in the GenBank database (as of December 2014). The lack of extensive transcriptomic and functional genomic resources is one of the major limiting factors of molecular research using non-model species. RNA-seq is a useful tool for studying differentially expressed transcripts in various tissues as well as developmental stages. Large-scale transcriptome sequencing of the tuberous roots of the sweet potato has facilitated gene discovery and has provided sequence resources for further molecular research (Schafleitner et al., 2010; Wang et al., 2010; Tao et al., 2012; Firon et al., 2013). Next-generation sequencing technology has also helped understand the molecular basis of replanting disease (Yang et al., 2011, 2014; Li et al., 2013). Moreover, it enables researchers to identify putative genes that might be involved in iridoid biosynthesis in *R*. *glutinosa* (Sun et al., 2012). However, the dynamic root transcriptome profile and the functional genes related to tuberous root development have not yet been explored. Therefore, the present study was conducted to improve our understanding of the molecular basis of tuberous root development in Rehmannia. We performed the first global analysis of the Rehmannia transcriptome during tuberous root development using the Illumina RNA-Seq method to identify genes related to sugar metabolism, hormone metabolism, and transcriptional regulation.

## Materials and methods

### Plant materials and RNA extraction

*R. glutinosa* plants (cultivar 85-5) were planted in pots containing a ratio of 1:1 vermiculite and humus soil, and were grown in a natural light-greenhouse. The root samples were collected at three stages: adventitious root (AR), thickening adventitious root (TAR), and the developing tuberous root (DTR), sampled at 30, 45, and 60 days after sprouting (DAS), respectively. For consistency, the roots were classified according to diameter: AR ≤ 2 mm, 3 mm ≤ TAR ≤ 5 mm, and 1 cm ≤ DTR ≤ 5 cm (Figure S1). Leaf and stem samples were collected at 60 DAS. Flower samples were collected during the floral season from 2-year-old plants. Five individual plants at various root developmental stages were collected and pooled together. Total RNAs were extracted using the Trizol™ reagent (Invitrogen, Carlsbad, CA, USA) and treated with DNase I (TaKaRa, Dalian, China).

### Illumina sequencing, *de novo* assembly, and functional annotation

To obtain a global view of the transcriptome that is relevant to tuberous root development in *R. glutinosa*, a reference transcriptome was built as described below. First, RNAs extracted from the ARs, TARs, and DTRs were equally pooled and sequenced on an Illumina HiSeq2000™ platform according to the manufacture's protocol. First-strand cDNA synthesis was performed by using random hexamer primers, whereas second-strand cDNA synthesis was conducted with buffer, dNTPs, RNaseH, and DNA polymerase I. These products were purified by using agarose gel electrophoresis and enriched by PCR to create the final cDNA library. The Beijing Genomics Institute commercially sequenced the cDNA library on an Illumina HiSeq2000™ platform, using paired-end 90 base pair (bp) reads to generate the raw sequences. The generated data are available at the NCBI Short Read Archive (http://www.ncbi.nlm.nih.gov/sra/SRX357355[accn]). Sequence assembly was conducted using the short reads assembling program SOAPdenovo, following the provided software instructions (Li et al., 2010). Second, the unique sequences produced by the SOAPdenovo pipeline were combined with the Rehmannia transcripts generated on the 454 GS FLX Titanium platform in a previous study (Sun et al., 2012) by using the CAP3 program (Huang and Madan, 1999). Assembly criteria included a 100-bp minimum match and a 95% minimum identity at the overlapping region. Third, all unigenes were then annotated by BLAST against various protein databases [i.e., Nr, SwissProt, Kyoto Encyclopedia of Genes and Genomes (KEGG), and Cluster of Orthologous Groups of proteins (COG)], with an *e*-value cutoff of 1e-5. All unigenes generated in the present study were subjected to Gene Ontology (GO) functional analysis. We used the Blast2GO program (Conesa and Götz, 2008) to identify GO terms for all assembled unigenes. Then, the WEGO software was used to perform GO functional classification for all unigenes to determine the distribution of gene functions of the species at the macromolecular level.

### RNA-seq quantification and KEGG pathway enrichment analysis

Three independent cDNA libraries prepared from three developmental staged roots (AR, TAR, and DTR) were subjected to RNA-Seq analysis. We generated 7.25 million (M), 7.13 M, and 7.50 M clean sequence reads for each cDNA library, with each read approximately 49-bp in length. The raw reads were deposited to the NCBI Short Read Archive (http://www.ncbi.nlm.nih.gov/Traces/sra/) under Accession numbers SRX700627, SRX700629, and SRX700631. The clean reads were then aligned to the reference transcriptome using SOAPaligner/soap2, allowing two-base mismatches. Reads that mapped to multiple locations were excluded from the analysis. Only unique matched reads were collected for statistical analysis of gene expression. The gene expression level of every unigene was calculated using the reads per kb per million reads (RPKM) method. The differentially expressed genes (DEG) in the ARs, TARs, and DTRs were analyzed as described elsewhere (Audic and Claverie, 1997), with some modifications. False discovery rate (FDR) was used to control the *p*-values. A threshold value of FDR ≤ 0.001 and an absolute value of log_2_Ratio ≥ 1 were used to judge the significance of the differences in gene expression. The DEGs were subjected to pathway enrichment analysis by comparing the DGE number (in a specific pathway) with that of the whole genome. The hypergeometric test was used to find significantly enriched pathways. The calculated *p*-value was submitted for Bonferroni correction, taking the corrected *p*-value ≤ 0.05 as threshold. Pathways fulfilling these criteria were defined as significantly enriched pathways.

### Identification of transcription factors (TFs) in *R. glutinosa*

Rehmannia TFs were searched and organized based on their sequence homology in PlantTFDB3.0 (http://planttfdb.cbi.pku.edu.cn/, Center for Bioinformatics, Peking University, China), which provides comprehensive information on various plant TF families (Jin et al., 2014).

### Quantitative real-time PCR

First-strand cDNA synthesis was performed with 500 ng of total RNA using the PrimeScript RT® reagent kit with gDNA Eraser (TaKaRa, Dalian, China), according to the manufacturer's instruction. The transcriptional profiles of unigenes were analyzed by qRT-PCR by using the SYBR® Premix Ex Taq II (TaKaRa, Dalian, China) and a real-time PCR system (Bio-Rad, USA). Gene-specific primers were designed by using the software Primer premier 5. The PCR conditions were as follows: a 95°C hold for 3 min; followed 95°C for 15 s, 58°C for 30 s, and 72°C for 20 s for a total of 40 cycles. Gene expression levels were normalized against the internal reference gene, *TIP41*. The relative expression ratio was calculated for each root sample relative to that of the ARs by using the 2^−ΔΔ*Ct*^ method.

### Quantification of endogenous plant hormones

Based on the significant changes in the levels of expression of hormone biosynthetic genes during root development, we examined the contents of plant hormones to determine their roles in tuberous root development. The levels of endogenous indole-3-acetic acid (IAA), gibberellic acid (GA_3_), zeatin riboside (ZR), and abscisic acid (ABA) were determined by using the method of Teng et al. (2006). The root samples were cleaned, weighed, flash frozen in liquid nitrogen, and stored at −80°C until analysis. The samples were ground in liquid nitrogen by using a mortar and pestle, extracted with ice-cold 80% methanol (v/v) containing 1 mol/L butylated hydroxytoluene to prevent oxidation, and then stored overnight at 4°C. The extracts were then centrifuged at 12,000 *g* for 15 min at 4°C. The residues were resuspended in the same ice-cold extraction solution and stored at 4°C for 1 h, and then centrifuged again at 12,000 *g* for 15 min at 4°C. The two resulting supernatants were combined and passed through a C-18 Sep-Pak cartridge (Waters, Milford, MA, USA). The efflux was collected and dried under nitrogen. The residues were then dissolved in 0.01 mol/L phosphate buffer solution (pH 7.4) and the concentrations of IAA, GA_3_, ZR, and ABA were determined by using enzyme-linked immunosorbent assay (ELISA), as desribed elsewhere (Teng et al., 2006). All the measurements were performed with three biological replicates for each development stage, and at least three samples were pooled together for each replicate.

## Results

### Establishment of a root reference transcriptome for rehmannia

We generated a total of 81,003 unique sequences from the cDNA library constructed from the ARs, TARs, and DTRs of Rehmannia by using the Illumina sequencing platform. We combined the 81,003 unique sequences with the transcripts generated by 454 GS FLX Titanium platform in a previous study by using the Cap3 program to increase the representativeness of the reference sequences. Our results identified a total of 93,172 unigenes with an average length of 383-bp that served as reference for subsequent analyses.

Alignment of unigenes with the nine full-length cDNA sequences resulted in eight significant hits in our assembly. These hits were well-covered by the unigenes (Supplementary Table S1), indicating that the assembly was well-represented at the genomic level and could be a useful source for gene discovery and global gene expression analysis.

### Functional annotation analysis

Gene annotation showed that of the 93,172 unigenes, 54,105 (58.07%) had significant matches in the public databases. The annotation ratio was similar to those of previous reports (Yang et al., 2011, 2014; Sun et al., 2012; Li et al., 2013). This transcriptome annotation of unigenes provides valuable information for the functional assignment of genes as well as for novel gene discovery.

**Figure 1:**
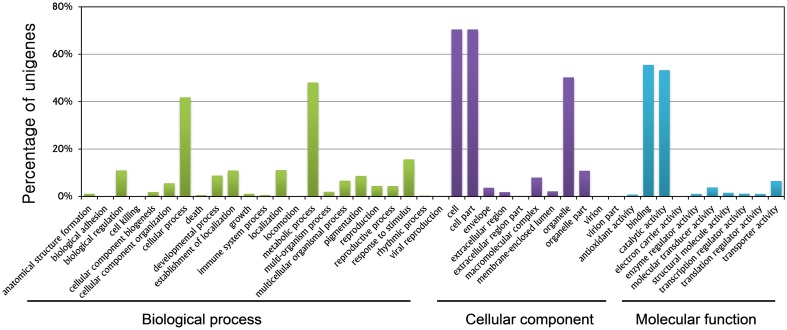
**Gene ontology classification of assembled genes**.

GO term assignment helped assign at least one GO term to a total of 24,428 unigenes. All GO terms were classified into 44 functional groups based on biological processes, cellular components, and molecular function categories. The GO analysis provided comprehensive information on unigene functions of *R*. *glutinosa*. Among the biological processes, unigenes assigned to metabolic (11,712, 47.94%) and cellular (10,172, 41.64%) processes were the most abundant. A high percentage of genes were grouped into the “response to stimuli” (3785, 15.49%), “biological regulation” (2646, 10.83%), and “developmental process” (2119, 8.67%) categories. These categories feature signal induction, perception, transduction, and modulation of functional genes, which are the main steps of plant root growth in response to environmental and nutrient stimuli, and eventually determine the root fate. These genes are potentially important topics that could be examined in research studies on root development (Figure 1, Supplementary Table S2).

**Figure 2:**
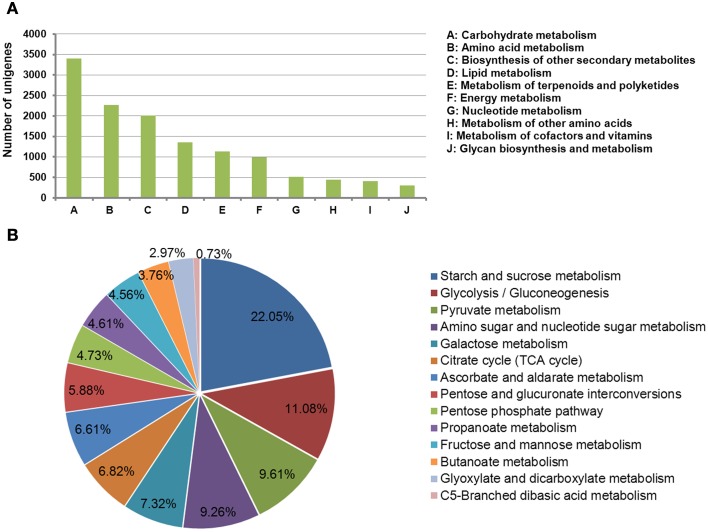
**Kyoto Encyclopedia of Genes and Genomes classification of assembled genes. (A)** Genes mapped to metabolic pathways; **(B)** genes involved in carbohydrate metabolism.

Metabolic pathway analysis assigned 23,171 significantly matched unigenes to the KEGG pathways. Among these, a total of 5644 unigenes were assigned to the metabolic pathways of carbohydrate metabolism, amino acid metabolism, biosynthesis of secondary metabolites, lipid metabolism, and metabolism of terpenoids and polyketides. The tuberous root stores energy (in the form of carbohydrates), as well as nutrients that are essential for growth and reproduction in the subsequent season. Carbohydrate metabolism was the most represented pathway during tuberous root development (Figure 2A). The unigenes involved in carbohydrate metabolism were further grouped into 12 sub-pathways. Among these, starch and sucrose metabolism, glycolysis/gluconeogenesis, pyruvate metabolism, amino sugar and nucleotide sugar metabolism, and galactose metabolism were the top five represented groups (Figure 2B).

### Analysis of differentially expressed genes

**Figure 3:**
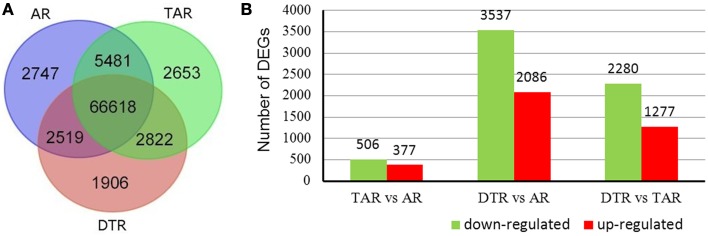
**Venn diagrams of unigenes of three libraries and statistical analysis of the differentially expressed genes (DEGs). (A)** Distribution of the unigenes of the three libraries; **(B)** The red columns indicate the upregulated DEGs and the green columns represent the downregulated DEGs in three pair-wise comparisons (FDR ≤ 0.001 and an absolute value of log_2_Ratio ≥ 1 was used as the significant threshold for DEGs).

The cDNA libraries from ARs, TARs, and DTRs, respectively generated 7.25, 7.13, and 7.50 M clean reads that were then aligned to the reference sequences to map 84,746 unigenes (Figure 3A). Most of these expressed genes (66,618 out of 84,746) were present in all three libraries. We used the RPKM method to calculate the gene expression values of each unigene in the three libraries. A pairwise comparison of the gene expression levels in the ARs, TARs, and DTRs revealed that there were a small number of DEGs between ARs and TARs. On the other hand, the comparison of AR/DTR revealed a high number of DEGs (Figure 3B). This result was in agreement with the root development sequence, i.e., TAR is developmentally close to AR. A total of 6794 unigenes were filtered as DEGs in the three pairwise comparisons (i.e., AR vs. TAR, AR vs. DTR, and TAR vs. DTR). The DEGs identified in the present study (Supplementary Table S3) were further used for gene enrichment analysis and identification of genes involved in tuberous root development.

To gain an overall view of the expression profiles of all DEGs across various developmental stages, cluster analysis was performed using the k-means method. Genes were divided into six groups based on their expression patterns (Figure S2). Clusters 1 and 2 comprised genes negatively or positively regulated during root development. Cluster 3 contained genes that were downregulated in the ARs. Cluster 4 was the most abundant group, with 2285 genes that were downregulated in DTRs. Cluster 5 consisted of genes that were downregulated in TARs and then upregulated in DTRs, and Cluster 6 consisted of genes that were upregulated in TARs and then downregulated in DTRs.

### Validation of RNA-seq-based gene expression analysis

**Figure 4:**
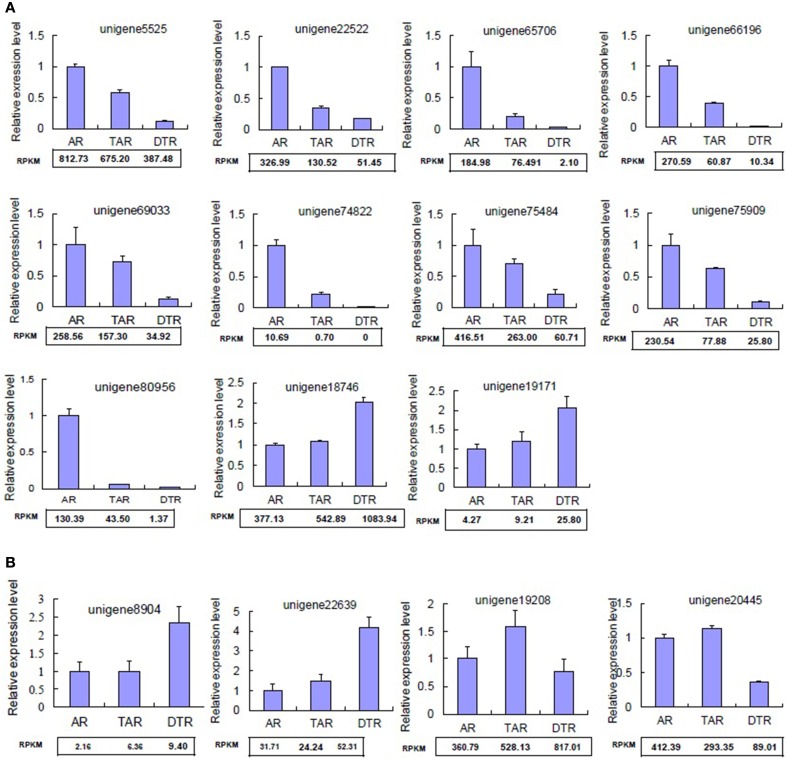
**Validation of DEGs by qRT-PCR. (A)** Genes displaying congruent results as revealed by qRT-PCR and RNA-seq analyses; **(B)** Genes showing inconsistent results revealed by qRT-PCR and RNA-seq analyses. The values indicate the means of three biological replicates ± SD. RPKM, reads per kb per million reads.

We randomly selected 15 differentially expressed unigenes for qRT-PCR validation of our RNA-seq data. The qRT-PCR profiles of 11 genes were completely in agreement with those obtained by using RNA-Seq (Figure 4A), although four differed at one or two stages (Figure 4B). Most of the congruent genes, including unigene5525, unigene66,196, and unigene75,484, were downregulated during tuberous root development (Figure 4A). Unigene5525 encodes a nodulin family protein. Recent advances highlight the importance of nodulin proteins in the transport of nutrients, solutes, amino acids, or hormones, as well as in major aspects of plant development in non-leguminous species (Denancé et al., 2014).

### Pathway enrichment analysis for differentially expressed genes

The results of our pathway enrichment analysis suggested that the tuberous root development involves the integration of several pathways. Phenylpropanoid biosynthesis, starch and sucrose metabolism, biosynthesis of plant hormones, and biosynthesis of terpenoids and steroids pathways were significantly affected by the functional transformation of the roots (Supplementary Table S4). Among these, it is notable that phenylpropanoid biosynthesis was downregulated throughout the developmental process. Genes related to lignin biosynthesis in the phenylpropanoid biosynthesis pathway such as cinnamate 4-hydroxylase (C_4_H), 4-coumarate CoA ligase (4CL), and cinnamyl alcohol dehydrogenase (CAD) were significantly downregulated in DTRs. Similar results were also observed in sweet potato (Firon et al., 2013), which suggested that lignification inhibits tuberous root formaion (Togari, 1950). However, changes in the biosynthesis of plant hormones, as well as that of terpenoids and steroids, were observed during the tuberous root enlargement stage (i.e., these pathways were enriched by DEGs, as indicated by the results of comparative analyses of DTR vs. TAR and DTR vs. AR).

### Candidate genes related to tuberous root development

#### Genes related to major carbohydrate metabolism

**Figure 5:**
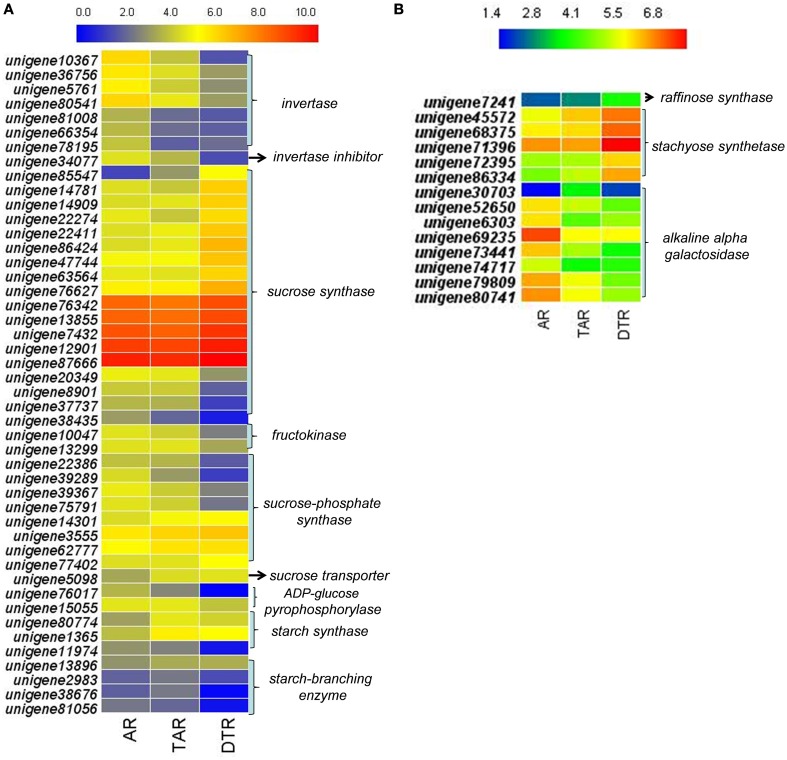
**Heat map of genes in ARs, TARs, and DTRs of *R*. *glutinosa***. The expression values were calculated by using the RPKM method and then were log2 transformed prior to the generation of heat maps. **(A)** The genes related to sucrose and starch metabolism; **(B)** key genes involved in stachyose metabolism.

We investigated the expression profiles of genes involved in starch and sucrose metabolism, glycolysis/gluconeogenesis, pyruvate metabolism, the citrate cycle (TCA cycle), and galactose metabolism to determine the molecular and biochemical changes in the levels of carbohydrates during root development. In higher plants, the entry of carbon from sucrose into cellular metabolism is catalyzed by either sucrose synthase (SUS) or invertase (INV). Interestingly, most of the transcripts encoding SUS were upregulated in DTRs when compared to ARs and TARs. On the other hand, the expression levels of the INV enzymes declined during root development. We did not find any transcript encoding sucrose-phosphatase, which converts sucrose-6-phosphate into sucrose. The transcripts encoding for sucrose-phosphate synthase showed either an upregulated or downregulated expression pattern during tuberous root development. Although two unigenes (unigene80,774 and 1365) that putatively encoded for starch synthase were upregulated in TARs and DTRs relative to that of the ARs, the enzyme ADP-glucose pyrophosphorylase (AGPase, a key enzyme in the starch biosynthetic pathway) was downregulated in DTRs. Moreover, the starch branching enzymes were downregulated during root development, suggesting that starch biosynthesis is not predominant in DTRs (Figure 5A). Most of the transcripts involved in glycolysis/gluconeogenesis, pyruvate metabolism, and TCA cycle were upregulated in ARs and TARs (data not shown), which suggests that added energy, reductants, and carbon building blocks for anabolism are required for root growth at AR and TAR stages.

**Figure 6:**
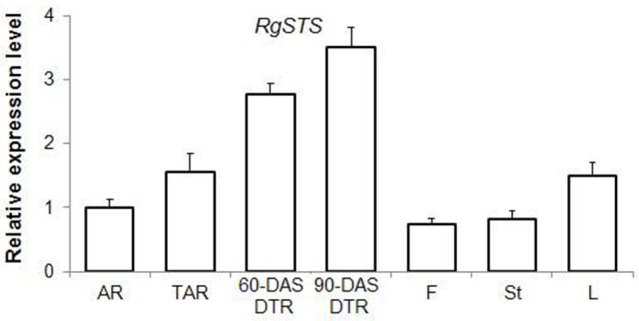
**The expression profile of** ***RgSTS*** **as revealed by qRT-PCR analysis**. AR, adventitious root; TAR, thickening adventitious root; 60-DAS DTR, developing tuberous root at 60 DAS; 90-DAS DTR, developing tuberous root at 90 DAS; F, flower; St, stem; L, leaf.

According to the KEGG, a total of 249 unigenes were mapped to the galactose metabolism pathway, which features raffinose family oligosaccharide (RFO) metabolism in *R*. *glutinosa*. Several unigenes in this category were involved in RFO metabolism, including transcripts encoding stachyose synthase (STS), raffinose synthase, and alkaline alpha-galactosidase, which are the key enzymes responsible for RFO degradation. We found five STS-encoding unigenes and one raffinose synthase-encoding unique in this category, which were enriched in DTRs (Figure 5B). A gene cloning experiment demonstrated that the five unigenes related to stachyose synthase corresponded to one full-length cDNA named *RgSTS* that is primarily expressed in tuberous roots (Figure 6).

### Genes involved in hormone metabolism

**Figure 7:**
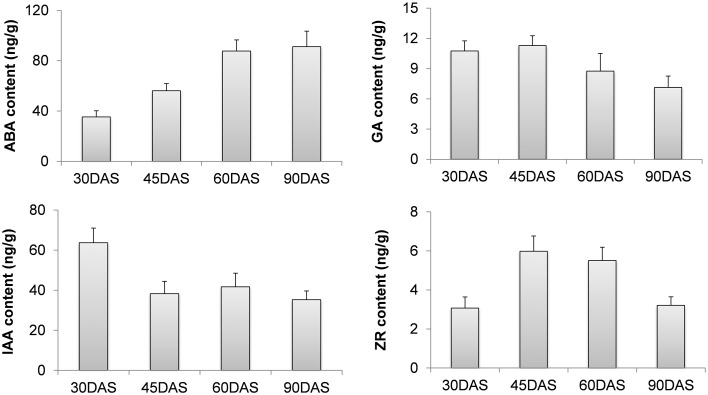
**Changes in the levels of various hormones during tuberous root development**.

The endogenous levels of phytohormones (IAA, ABA, ZR, and GA_3_) were measured in four root stages, which included 30-DAS ARs, 45-DAS TARs, 60-DAS DTRs, and 90-DAS DTRs. The ABA concentration increased during tuberous root development, reaching a maximum at 90 DAS, whereas GA content decreased during root development. The levels of IAA and ZR fluctuated during root development. In general, the highest IAA level was observed in ARs, whereas the ZR level increased at initial stages of root thickening (45 and 60 DAS) and then declined at 90 DAS (Figure 7). Based on the *Arabidopsis* Hormone Database (http://ahd.cbi.pku.edu.cn) (Jiang et al., 2011) and the KEGG annotation, we identified 82 putative genes related to ABA, GA, IAA, ZR, and ethylene metabolism. Most of these genes were homologs of *Arabidopsis* genes associated with various hormone actions (Jiang and Guo, 2010). The most significant were the genes crucial for biosynthesis of ABA, GAs, and cytokinins such as *9-cis-epoxycarotenoid dioxygenase, gibberellin 20-oxidas*e, *adenylate isopentenyltransferase*, and *cytokinin hydroxylase* (*CYP735A*) (Table 1). Besides genes involved in hormone biosynthesis, we also identified several genes responsible for hormone catabolism and conjugation, which function in hormone balance. We did not detect the hormone ethylene in our samples. However, we identified ethylene biosynthetic genes including *S-adenosylmethionine synthase, 1-aminocyclopropane-1-carboxylate oxidase*, and *1-aminocyclopropane-1-carboxylate synthase* (Table 1). The expression patterns of the ethylene biosynthetic genes showed that ethylene content increased during tuberous root development (Table 1).

**Table 1:** **Candidate genes involved in hormone metabolism**.

**Gene ID**	**Description**	**RPK**			**Homology**	***E*-value**
		**AR**	**TAR**	**DTR**		
**ABA METABOLISM**						
Unigene14,731	*Zeaxanthin epoxidase*	2.62	4.40	7.35	AT5G67030	2.0E-118
Unigene79,683	*Zeaxanthin epoxidase*	2.90	6.09	6.10	AT5G67030	4.0E-60
Unigene71,988	*9-Cis-epoxycarotenoid dioxygenase NCED3*	19.40	38.85	57.98	AT3G14440	1.0E-31
Unigene77,467	*9-Cis-epoxycarotenoid dioxygenase NCED3*	5.68	11.12	24.52	AT3G14440	6.0E-78
Unigene250	*9-Cis-epoxycarotenoid dioxygenase NCED3*	11.92	30.38	32.01	AT3G14440	2.0E-54
Unigene36,249	*9-Cis-epoxycarotenoid dioxygenase NCED3*	14.09	10.66	6.33	AT3G14440	2.0E-38
Unigene38,689	*9-cis-epoxycarotenoid dioxygenase NCED3*	5.48	9.55	0.60	AT3G14440	3.0E-133
Unigene50,527	*9-Cis-epoxycarotenoid dioxygenase NCED3*	0.00	11.82	4.44	AT3G14440	2.0E-25
Unigene71,307	*9-Cis-epoxycarotenoid dioxygenase NCED3*	4.36	2.57	1.71	AT3G14440	8.0E-35
Unigene71,988	*9-Cis-epoxycarotenoid dioxygenase NCED3*	19.40	38.85	57.98	AT3G14440	3.0E-30
Unigene76,271	*9-Cis-epoxycarotenoid dioxygenase NCED3*	7.63	5.61	8.74	AT3G14440	4.0E-69
Unigene79,090	*Probable 9-cis-epoxycarotenoid dioxygenase NCED5*	5.07	4.96	5.43	AT1G30100	4.0E-93
Unigene8865	*Probable 9-cis-epoxycarotenoid dioxygenase NCED5*	4.91	13.95	1.45	AT1G30100	2.0E-37
Unigene14,196	*Abscisic-aldehyde oxidase AAO3*	13.46	26.37	47.13	AT2G27150	2.0E-47
Unigene18,426	*Abscisic-aldehyde oxidase AAO3*	32.73	55.69	127.14	AT2G27150	4.0E-80
Unigene34,517	*Abscisic-aldehyde oxidase AAO3*	12.29	26.27	44.96	AT2G27150	1.0E-33
Unigene67,269	*Abscisic-aldehyde oxidase AAO3*	25.54	56.07	86.28	AT2G27150	3.0E-34
Unigene70,327	*Abscisic-aldehyde oxidase AAO3*	23.75	42.07	69.06	AT2G27150	2.0E-27
Unigene38,636	*Abscisic acid 8′-hydroxylase 2*	30.57	14.30	4.09	AT2G29090	7.0E-36
Unigene91,159	*Abscisic acid 8′-hydroxylase 2*	888.13	563.73	316.29	AT2G29090	2.0E-49
Unigene1492	*Abscisic acid 8′-hydroxylase 3*	733.53	468.97	259.24	AT5G45340	5.0E-56
Unigene5332	*Abscisic acid 8′-hydroxylase 3*	1311.82	854.60	504.79	AT5G45340	2.0E-17
Unigene6417	*Abscisic acid 8′-hydroxylase 3*	392.57	248.49	157.31	AT5G45340	4.0E-30
Unigene7080	*Abscisic acid 8′-hydroxylase 3*	264.93	182.89	118.90	AT5G45340	5.0E-27
Unigene83,652	*Abscisic acid 8′-hydroxylase 3*	324.54	189.23	122.69	AT5G45340	1.0E-21
Unigene84,444	*Abscisic acid 8′-hydroxylase 3*	112.50	67.96	46.91	AT5G45340	1.0E-15
Unigene93,110	*Abscisic acid 8′-hydroxylase 3*	505.21	349.04	199.26	AT5G45340	3.0E-79
**IAA METABOLISM**						
Unigene75,195	*Probable indole-3-pyruvate monooxygenase YUC1*	11.81	8.85	4.09	AT4G32540	1.0E-21
Unigene1874	*Probable indole-3-pyruvate monooxygenase YUC3*	16.31	11.63	6.31	AT1G04610	1.0E-39
Unigene28,174	*Probable indole-3-pyruvate monooxygenase YUC3*	0.0	4.71	1.89	AT1G04610	9.0E-17
Unigene74,792	*Tryptophan N-monooxygenase 1*	13.58	23.11	4.91	AT4G39950	1.0E-28
Unigene5206	*CYP83B1*	33.87	74.68	32.62	AT4G31500	1.0E-48
Uunigene14,470	*Indole-3-acetic acid-amido synthetase GH3.2*	45.53	37.57	9.15	AT4G37390	8.0E-102
Unigene14,718	*Indole-3-acetic acid-amido synthetase GH3.2*	145.55	90.56	21.78	AT4G37390	3.0E-58
Unigene17,094	*Indole-3-acetic acid-amido synthetase GH3.2*	36.03	13.56	5.62	AT4G37390	2.0E-107
Unigene22,275	*Indole-3-acetic acid-amido synthetaseGH3.2*	21.87	9.34	2.20	AT4G37390	2.0E-40
Unigene61,121	*Indole-3-acetic acid-amido synthetaseGH3.2*	39.58	30.56	4.71	AT4G37390	2.0E-29
Unigene11,653	*Indole-3-acetic acid-amido synthetase GH3.1*	286.50	179.93	50.52	AT1G59500	5.0E-41
Unigene75,484	*Indole-3-acetic acid-amido synthetase GH3.1*	416.51	263.00	60.71	AT1G59500	1.0E-53
Unigene15,733	*Indole-3-acetic acid-amido synthetase GH3.5*	72.20	30.25	5.38	AT4G27260	1.0E-53
Unigene31,393	*Indole-3-acetic acid-amido synthetase GH3.5*	116.82	46.80	5.86	AT4G27260	4.0E-103
Unigene50,368	*Indole-3-acetic acid-amido synthetase GH3.5*	232.14	162.49	35.52	AT4G27260	1.0E-18
Unigene76,234	*Indole-3-acetic acid-amido synthetase GH3.5*	88.12	35.67	4.39	AT4G27260	7.0E-73
**ETHYLENE BIOSYNTHESIS**						
Unigene14,237	*1-Aminocyclopropane-1-carboxylate oxidase ACO1*	41.74	19.09	30.05	AT2G19590	3.0E-47
Unigene5632	*1-Aminocyclopropane-1-carboxylate oxidase ACO1*	123.43	49.61	90.48	AT2G19590	3.0E-60
Unigene19,668	*1-Aminocyclopropane-1-carboxylate synthase ACS2*	13.55	32.58	83.44	AT1G01480	1.0E-24
Unigene6520	*1-Aminocyclopropane-1-carboxylate synthase ACS2*	31.43	61.59	150.87	AT1G01480	6.0E-21
Unigene19,547	*1-Aminocyclopropane-1-carboxylate synthase ACS2*	39.23	66.60	148.33	AT4G11280	8.0E-100
Unigene1475	*S-adenosylmethionine synthase 1*	202.37	183.91	44.41	AT1G02500	3.0E-33
Unigene29,162	*S-adenosylmethionine synthase 1*	72.36	61.30	23.68	AT1G02500	1.0E-61
Unigene49,524	*S-adenosylmethionine synthase 1*	215.76	200.86	102.90	AT1G02500	2.0E-28
Unigene84,080	*S-adenosylmethionine synthase 1*	220.69	196.18	97.51	AT1G02500	6.0E-32
**GA METABOLISM**						
Unigene59,753	*Gibberellin 20-oxidase 3*	4.97	3.65	1.22	AT5G07200	8.0E-16
Unigene71,686	*Gibberellin 20-oxidase 2*	6.00	0.84	1.68	AT5G51810	4.0E-33
Unigene78,509	*Gibberellin 3-beta-dioxygenase 2*	7.56	4.45	2.97	AT1G80340	5.0E-64
Unigene79,622	*Gibberellin 2-beta-dioxygenase 3*	37.67	32.82	7.40	AT1G30040	7.0E-74
Unigene65,647	*Gibberellin 2-beta-dioxygenase 3*	28.87	30.39	4.20	AT2G34555	1.0E-16
Unigene13,828	*Gibberellin 2-beta-dioxygenase 3*	49.05	58.82	10.77	AT2G34555	3.0E-20
**CYTOKININ METABOLISM**						
Unigene32,822	*Adenylate isopentenyltransferase 5*	7.26	9.78	0.0	AT5G19040	9.0E-32
Unigene27,285	*Adenylate isopentenyltransferase 3*	9.07	13.33	3.71	AT3G63110	1.0E-37
Unigene32,812	*Adenylate isopentenyltransferase 3*	4.04	10.31	0.79	AT3G63110	2.0E-25
Unigene38,121	*Adenylate isopentenyltransferase 3*	3.14	3.08	0.44	AT3G63110	5.0E-31
Unigene80,851	*Adenylate isopentenyltransferase*	5.55	10.31	0.86	osa:4334529	9.0E-23
Unigene34,535	*Cytokinin hydroxylase/CYP735A2*	20.28	35.93	6.55	AT1G67110	8.0E-38
Unigene37,550	*Cytokinin hydroxylase/CYP735A2*	8.25	6.57	1.52	AT1G67110	4.0E-29
Unigene37,699	*Cytokinin hydroxylase/CYP735A2*	5.69	3.72	2.79	AT1G67110	5.0E-51
Unigene79,424	*Cytokinin hydroxylase/CYP735A2*	8.67	17.43	5.54	AT1G67110	2.0E-46
Unigene39,497	*Cytokinin hydroxylase/CYP735A1*	15.65	28.11	39.20	AT5G38450	4.0E-75
Unigene79,648	*Cytokinin hydroxylase/CYP735A1*	15.42	20.01	2.46	AT5G38450	2.0E-25
Unigene31,666	*Cytokinin hydroxylase/CYP735A1*	13.66	10.80	10.82	AT5G38450	7.0E-36
Unigene78,310	*Cytokinin hydroxylase/CYP735A1*	3.10	5.06	1.52	AT5G38450	9.0E-22
Unigene2280	*tRNA dimethylallyltransferase*	7.92	14.23	14.26	vvi:100251208	3.0E-19
Unigene47,050	*tRNA dimethylallyltransferase*	1.62	4.77	1.59	vvi:100245121	5.0E-21
Unigene74,617	*tRNA dimethylallyltransferase*	1.45	1.42	3.55	vvi:100245121	2.0E-34
Unigene22,069	*Cytokinin dehydrogenase 7/CKX7*	13.42	11.03	2.41	AT5G21482	0.0
Unigene714	*Cytokinin dehydrogenase 9/CKX9*	133.47	163.53	37.58	osa:4338605	4.0E-180
Unigene69,433	*Cytokinin dehydrogenase 5/CKX5*	0.94	4.63	0.0	AT1G75450	1.0E-41
Unigene75,186	*Cytokinin-N-glucosyltransferase UGT76C1*	30.57	53.79	59.36	AT5G05870	3.0E-24
Unigene36,757	*Cytokinin-N-glucosyltransferase UGT76C2*	26.07	31.32	11.56	AT5G05860	2.0E-09
Unigene18,128	*Cytokinin-N-glucosyltransferase UGT76C2*	8.03	17.72	36.82	AT5G05860	1.0E-12
Unigene3995	*Cytokinin-N-glucosyltransferase UGT76C2*	26.67	36.59	80.06	AT5G05860	5.0E-23
Unigene9623	*Cytokinin-N-glucosyltransferase UGT76C2*	38.20	60.29	100.12	AT5G05860	1.0E-82

### TFs

We identified 2059 TFs through a BLAST search against the Plant Transcription Factor Database 3.0 (http://planttfdb.cbi.pku.edu.cn) (Jin et al., 2014). These TFs accounted for 2.21% (2059/93,172) of the total sequences. We classified the TFs in *R*. *glutinosa* into 55 families according to the Plant TFDB category system. Of these, bHLH (8.35%), C_3_H (6.31%), MYB (5.93%), WRKY (5.73%), and C_2_H_2_ (5.39%) were the most abundant types. The distribution of TFs across the genome of *R*. *glutinosa* was similar to those of *Arabidopsis* and rice, except that a higher number of C_3_H and GRAS family TFs were present in *R*. *glutinosa* as compared with that of *Arabidopsis* and rice (Supplementary Table S5). Among these, a total of 267 were identified as DEGs. The bHLH (34, 12.73%), WRKY (23, 8.61%), GRAS (23, 8.61%), ERF (21, 7.87%), MYB (16, 5.99%), C_3_H (15, 5.62%), G2-like (15, 5.62%), and HD-ZIP (11, 4.12%) were the top differentially expressed categories; the remaining categories contained <10 differentially expressed TFs (Supplementary Table S5).

We also determined the expression profiles of the differentially expressed TFs. Most transcripts in the C_3_H, GRAS, and ERF families were upregulated in DTRs compared to that of ARs and TARs, whereas the expression of most G2-like and ARF transcripts was downregulated in DTRs (Supplementary Table S6). Interestingly, the majority of the transcripts encoding ARF proteins were upregulated in ARs and TARs. The expression levels of these transcripts were positively correlated with IAA content, suggesting that ARFs played a role in AR formation (Supplementary Table S6).

## Discussion

### A first insight into tuberous root development in *R. glutinosa*

Tuberous roots of species such as sweet potato, cassava, *R*. *glutinosa*, and *Polygonum multiflorum* have significant medicinal value, in addition to being an important food resource to humans. Despite their economic importance, research on tuberous roots has not received the same attention as the root systems of model plant species. Lack of extensive genomic sequences and information on functional genes, especially gene expression profiles and functional assignment of genes, has hindered our understanding of the molecular processes of tuberous root development in related species. In the present study, the application of the RNA-Seq technology generated a total of 93,172 transcriptome sequences. These sequences provide a valuable resource for root specific gene discovery and for the assembly of a Rehmannia reference at the genomic level. Our data, which was generated from developing roots, may facilitate in the analysis of transcriptomic changes associated with root development. Moreover, it may help in the genetic manipulation of varieties to control the development of tuberous roots. The global gene expression profiles of ARs, TARs, and DTRs (Supplementary Table S3) revealed the dynamic characteristics of the developing root transcriptome. This RNA-seq based transcriptome analysis provides an initial view of the tuberous root development process, assisting in the identification of candidate genes related to hormone biosynthesis, sugar metabolism, and TFs, thus paving the way for further elucidation of the molecular mechanism underlying tuberous root development.

### The relationship of sugars and tuberous root development

Glucose and sucrose control various developmental events such as seed germination, flowering, and senescence, and modulate organ formation. Sucrose affects the formation of sink organs and is a notable inducer of tuberization in potato (Perl et al., 1991) and yam (Ovono et al., 2009). Similarly, a high sucrose concentration is required for the *in vitro* induction of tuberous roots in *R*. *glutinosa* (Xue et al., 2002). An exogenous supply of sucrose enhances the production of sweet potato tuberous roots (Tsubone et al., 2000). Sucrose cleavage functions in the allocation of crucial carbon resources, as well as in the initiation of hexose-based sugar signals in importing structures, which have profound developmental effects (Koch, 2004). The channeling of sucrose into a different pathway requires its cleavage. The isoforms of INV and SUS cleave sucrose to channel it into different pathways (Sturm and Tang, 1999). INVs are crucial for sink initiation and expansion, whereas SUSs are mainly responsible for reserve storage and organ maturation (Koch, 2004). The present study has shown that most of the unigenes encoding for SUS were upregulated in DTRs as compared to that of the ARs and TARs; a few were downregulated in DTRs, which may represent the different isoforms of SUS. In contrast, INVs were downregulated during root development. Similar results were observed in sweet potato (Li and Zhang, 2003; McGregor, 2006). The expression profile of INVs and SUSs indicate that these sequentially contribute to the development of tuberous roots. The mechanism underlying how sugars regulate the gene activity of the enzymes related to tuberous root development, including the sucrose cleaving enzymes, remains unclear. Moreover, the crosstalk between sugar and hormone signaling pathways in modulating tuberous root architecture has yet to be determined. Sugars are active effectors of multiple processes, including the biosynthesis and perception of hormones such as ABA (Rolland et al., 2002; Koch, 2004). Recent studies have shown that sugar and hormone signals are tightly coupled to each other during plant growth and development (Gazzarrini and McCourt, 2001; Gibson, 2005; Eveland and Jackson, 2012). The present study identified one transcript that encoded for a sucrose transporter (unigene5098) and another transcript encoding for an INV inhibitor (unigene34,077).

Previous studies have shown that the expression of AGPase and starch-branching enzymes are upregulated in tuberous roots of sweet potato (McGregor, 2006; Wang et al., 2006). However, both enzymes were downregulated during root development in *R*. *glutinosa* (Figure 5A). These differences can be attributed to the fact that sweet potato accumulates starch, whereas *R*. *glutinosa* stores stachyose in tuberous roots. The metabolism and physiological function of stachyose during tuberous root development are not well-understood. RFOs are transportable carbohydrates found in seeds, tubers, and leaves (Handley et al., 1983; Keller and Matile, 1985; Horbowicz and Obendorf, 1994; Bachmann and Keller, 1995). These are regarded as reserve carbohydrates for seedling germination; these also influence seed viability, as well as contribute to tolerance to various stresses (Bachmann et al., 1994; Horbowicz and Obendorf, 1994; Obendorf, 1997). In the present study, the tuberous root-specific expression of *RgSTS* suggested that stachyose functions as a reserve carbohydrate that provides energy and the carbon skeleton for plant propagation in favorable conditions, rather than serve as a transportable and osmotic sugar. *Stachys sieboldii* stores a high amount of stachyose in their tubers, which significantly decreases during the sprouting period (Keller and Matile, 1985), thus suggesting its role in energy storage. Transgenic experiments have demonstrated that reserve starch is not necessary for tuber formation in potato (Müller-Röber et al., 1992); therefore, stachyose might also not be a prerequisite for tuber root formation in Rehmannia. Transgenic Rehmannia lines will help elucidate the physiological role of stachyose in tuberous root formation.

### Hormones, hormone-related genes, and tuberous root development

Our experiments showed that ABA levels increased during tuberous root development. Furthermore, the expression patterns of most unigenes encoding 9-cis-epoxycarotenoid dioxygenase (*NCED*) and all the sequences encoding abscisic-aldehyde oxidase (*AAO*) were positively correlated with changes in the level of ABA during root development. The endogenous levels of ABA show a positive correlation with the thickening potential of sweet potato tuberous roots (Nakatani et al., 1988). Therefore, ABA might play a crucial role in tuberous root enlargement and/or the sink potential establishment. Recent studies (Arenas-Huertero et al., 2000; Cheng et al., 2002; Travaglia et al., 2007) have implicated ABA in plant growth and carbohydrate accumulation, in addition to its function as a stress hormone. Endogenous ABA levels promote plant growth and development in the absence of severe stress (Arenas-Huertero et al., 2000; Cheng et al., 2002). Exogenous application of ABA to wheat leaves increases yield by accumulating carbohydrates and redistributing grains (Travaglia et al., 2007). Therefore, ABA plays a complex physiological role in plant growth and development.

In sweet potato, high cytokinin and auxin levels promote the onset and subsequent primary thickening and growth of tuberous roots (Matsuo et al., 1983, 1988; Nakatani and Komeichi, 1991, 1992). The present study showed that the level of endogenous ZR increased in 45-DAS TARs and 60-DAS DTRs, indicating that ZR promotes tuberous root formation and its initial enlargement. The expression levels of *adenylate isopentenyltransferase*s (*IPTs*) and *cytokinin hydroxylases* (*CYP735As*) increased in 45-DAS TARs, but dramatically declined in 60-DAS DTRs, which was not in agreement with the ZR contents. Auxin has been shown to control AR formation (Geiss et al., 2009; Li et al., 2009b). Hormone content analysis conducted in the present study has confirmed the role of auxin in AR formation, as AR has the highest IAA content in all the tested roots. Previous studies have implicated multiple pathways in the *de novo* biosynthesis of auxins; however, the details have yet to be elucidated (Mano and Nemoto, 2012). We identified transcripts encoding the enzymes tryptophan N-monooxygenase and indole-3-pyruvate monooxygenase, which are involved in auxin biosynthesis. However, their expression patterns did not coincide with the IAA profiles. Both genes showed a high expression level in ARs compared to that in DTRs. In the present study, ZR and IAA contents were not consistent with the levels of their biosynthetic genes, implicating the complexity of their regulation. GA_3_ levels declined during root development and were corroborated by the expression studies of *gibberellin 20-oxidase* and *gibberellin 3-beta-dioxygenase*, which also declined during the process. The effects of GA on tuberous root have not been fully determined. GA promotes stolon elongation and inhibits tuber formation in potato (Smith and Rappaport, 1960; Kumar and Wareing, 1972; Railton and Wareing, 1973; Krauss and Marschner, 1982). Tubers and tuberous roots are of different origins; however, these may recruit common physiological factors such as hormones to trigger downstream actions when initiating sink development. Several GRAS-domain containing TFs were upregulated during root development (Supplementary Table S6). GRAS proteins such as DELLA and SCARECROW are essential components of the GA signaling pathway and function in multiple plant process, including root development (Bolle, 2004; Hirsch and Oldroyd, 2009). Ethylene biosynthetic genes and ERFs were also upregulated during tuberous root development, suggesting a role for ethylene in the process. Similar results were reported in sweet potato tuberous roots (Firon et al., 2013). ERFs control cell dedifferentiation, metabolite biosynthesis, and trait development (Song et al., 2013). The findings of the present study may facilitate in better understanding the role of ethylene in tuberous root development.

We also identified various genes involved in hormone homeostasis, which include abscisic acid 8′-hydroxylase, gibberellin 2-beta-dioxygenase, GH3, cytokinin hydroxylase, cytokinin dehydrogenase, and UDP-glycosyltransferase 76C2. Among these, GH3 catalyzes the synthesis of IAA-amino acid conjugates, thus providing a mechanism for the plant to cope with the presence of excess auxin (Staswick et al., 2005). A recent report demonstrated that auxin-inducible proteins GH3.3, GH3.5, and GH3.6, which can conjugate JA, do not act by regulating auxin levels, but control JA homeostasis in Arabidopsis. Thus, auxin controls AR initiation through a complex network that requires GH3, which regulates JA homeostasis that ultimately controls AR formation (Gutierrez et al., 2012). The Arabidopsis study (Gutierrez et al., 2012) can partially explain the abundance of GH3 genes in ARs, which have high IAA content. Auxin promotes root growth by modulating the gibberellin response (Fu and Harberd, 2003). Moreover, ethylene (perhaps via an auxin-dependent pathway) and auxin both alter the abundance/stability of DELLA (Achard et al., 2003). Hormones also interact with other signals to regulate plant development. Sugars and ABA tend to act synergistically during embryo growth, in transitioning from a phase of rapid cell division to cell enlargement, and in the accumulation of storage reserves (Eveland and Jackson, 2012). Therefore, a complex, dynamic, and possibly interconnected signaling network is involved in tuberous root development. Our results may help determine the role of hormones in tuberous root development at the molecular level.

### Overview of the biological processes related to tuberous root development

Tuberous root formation and enlargement are of two vital important processes of Rehmannia tuberous root development. However, the root development programs in *R. glutinosa* are highly complex and need to be finely controlled and coordinated by the intervention of several crosstalks. Based on the data presented in the present study, we propose a preliminary overview of the important biological processes occurring during Rehmannia root development, which are in part schematically represented in Figure S3. In favorable conditions, including environment factors such as light, temperature, humidity, and nutrient status, the plants detect these signals and initiate various developmental processes such as root development, which involves hormone regulation (including hormone biosynthesis, conjugation, transport, and degradation), carbohydrate metabolism, and upregulation of a series of root development-related genes. In general, at the AR formation stage, the level of the plant hormone auxin significantly increases in ARs, which has been shown to be intimately involved in the process of adventitious rooting (Geiss et al., 2009; Li et al., 2009b). Crosstalks between auxin and the plant hormones GA and JA finely tune the adventitious rooting process (Fu and Harberd, 2003; Gutierrez et al., 2012). Once the carbohydrate supply in the whole plant is sufficient, ARs transform into tuberous roots under high cytokinin concentrations, which has been implicated in the onset of tuberous root in sweet potato (Matsuo et al., 1983, 1988; Nakatani and Komeichi, 1991). Then, the tuberous roots become the largest sinks; the plants trigger a series of biological processes such as ABA and ethylene biosynthesis, conversion of sugars, which act as developmental signals to regulate the transport and metabolism of carbohydrates, and the transcription of developmental genes in DTRs. As earlier described, certain crosstalks between the hormone and sugar signaling pathways might be involved in the regulation of tuberous root development. Tuberous root development is accompanied by the downregulation of genes in the lignin biosynthesis pathway. The degree of lignification in roots might be a limiting factor for tuberous root formation and enlargement, as these were also observed in sweet potato (Togari, 1950; Firon et al., 2013).

## Conclusions

In the present study, we generated the root transcriptome of *R*. *glutinosa*. We compared the expression profiles of ARs, TARs, and DTRs to discover a series of genes involved in tuberous root development. Our study provides valuable insights into the molecular basis of tuberous root development and presents critical information for breeding and engineering that may yield large amount of Rehmannia varieties. We also showed that tuberous root development is a complicated process that involves the interaction of several genes and various physiological factors. Our large transcriptome dataset also provides information that could be employed in comparative analysis studies of closely related species.

### Conflict of interest statement

The authors declare that the research was conducted in the absence of any commercial or financial relationships that could be construed as a potential conflict of interest.
