# Supplementary material for: Dynamic transcriptional profiling provides insights into tuberous root development in Rehmannia glutinosa
Source: Front Plant Sci. 2015 Jun 10;6:396. doi: 10.3389/fpls.2015.00396 (PMC4461823; doi:10.3389/fpls.2015.00396)
Supplement: Supplementary file 2 [file Image1.PDF]

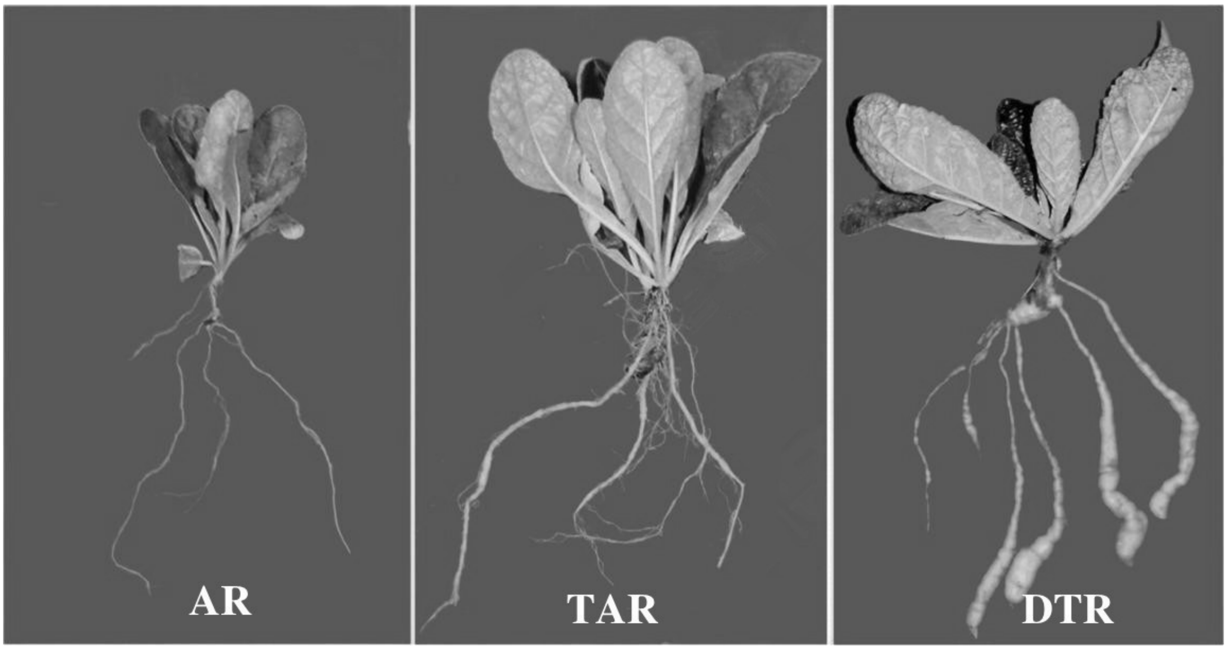

Supplemental Figure 1. Representative Rehmannia plants at AR, TAR and DTR stages, corresponding to 15, 30, and 60 days after sprouting.

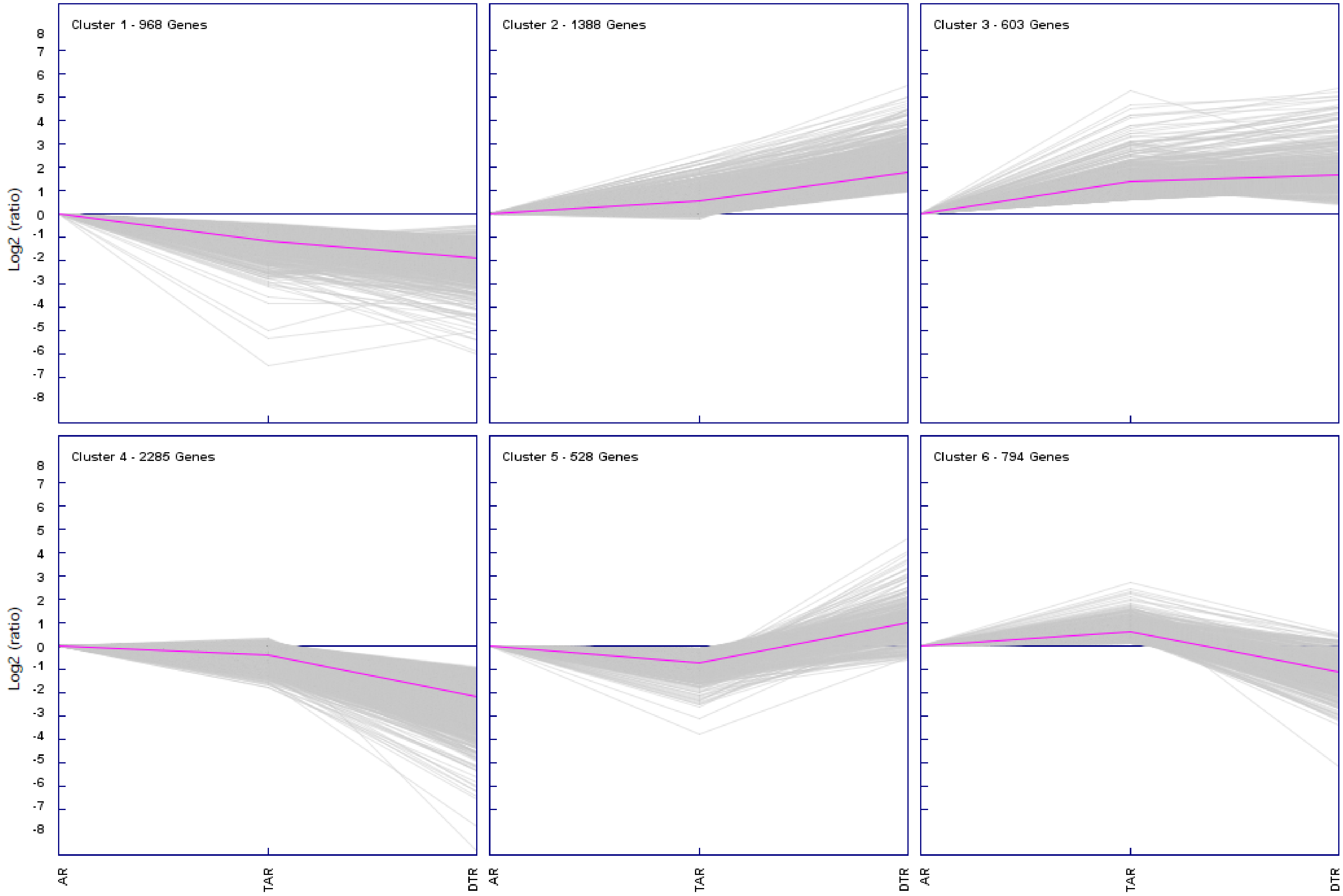

Supplemental Figure 2. Clustering of differentially expressed genes (DEGs). The six major clusters obtained by K-means algorithm, representing genes that are consistently downregulated (1), upregulated (2), and stage-specifically regulated (3,4,5,6). The y-axis represents the log2 ratio of RPKM of genes at each stage relative to that observed in ARs.

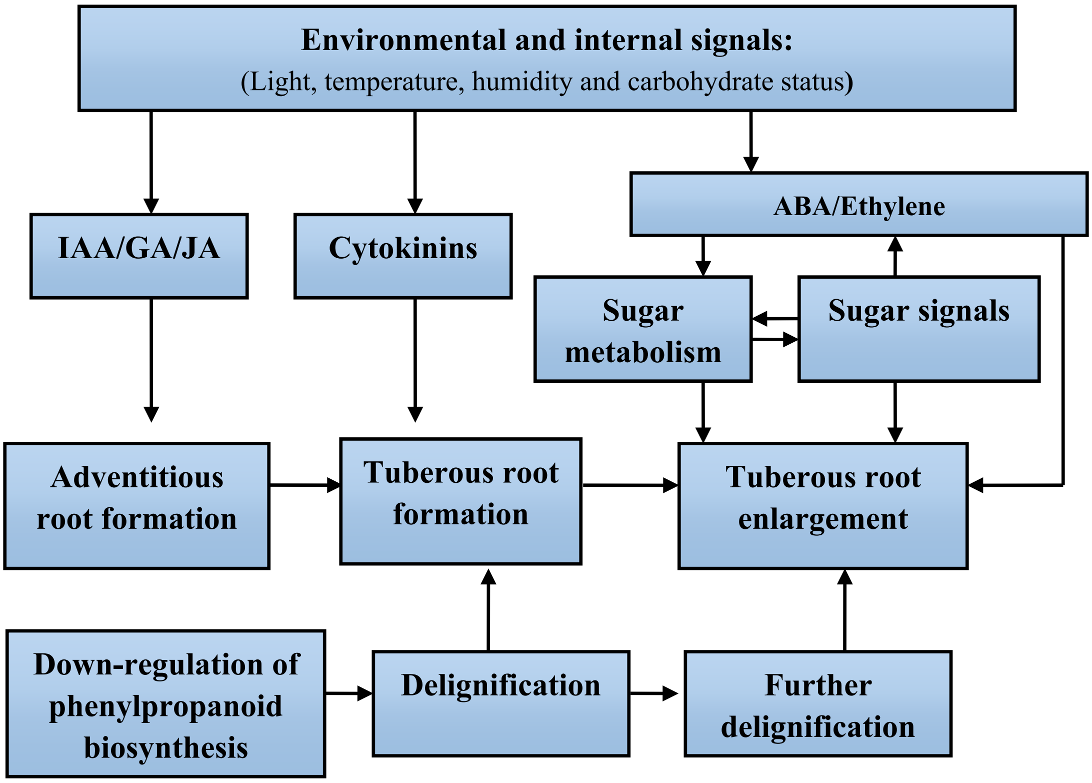

Supplemental Figure 3, Overview for the biological processes during Rehmannia tuberous root development.
